# The Role of Within-Person Variability in Forming Stable Face Representations

**DOI:** 10.1068/ig3

**Published:** 2013-10-01

**Authors:** S Andrews, A M Burton

**Affiliations:** University of Aberdeen, UK; University of Aberdeen, UK

## Abstract

We are usually able to recognise novel instances of familiar faces with little difficulty; yet unfamiliar face recognition can be dramatically impaired by natural within-person variability. Unless otherwise prompted, naturally varying instances of an unfamiliar face are often perceived as belonging to multiple different people (Jenkins, White, Van Montford & Burton, 2011). In Experiment 1 participants sorted naturally varying images of unfamiliar faces into their separate identities; half were told that only two target identities were present (constrained), while half were given no indication of the number of targets (unconstrained). Results indicate that unconstrained participants sorted images into 7.5 identities (mean 5 images per pile). On a subsequent matching test, participants who had performed a constrained sort were more accurate than unconstrained sorters, although matching task accuracy was greater for identities seen in the sorting task than for completely novel faces. To investigate this form of learning, Experiment 2 replicated the design using equally complex non-face stimuli (photographic negative faces from Experiment 1). Results indicate that while sorting and matching accuracy was generally poorer, matching task accuracy was greater for learnt than novel negatives. The implications of these findings are discussed with regards to the importance of within-person variability in developing stable face representations.

